# Correlational analysis for identifying genes whose regulation contributes to chronic neuropathic pain

**DOI:** 10.1186/1744-8069-5-7

**Published:** 2009-02-19

**Authors:** Anna-Karin Persson, Mathias Gebauer, Suzana Jordan, Christiane Metz-Weidmann, Anke M Schulte, Hans-Christoph Schneider, Danping Ding-Pfennigdorff, Jonas Thun, Xiao-Jun Xu, Zsuzsanna Wiesenfeld-Hallin, Ariel Darvasi, Kaj Fried, Marshall Devor

**Affiliations:** 1 Center for Oral Biology, Novum, Karolinska Institutet, S-141 04 Huddinge, Sweden; 2 Discovery Research, Sanofi-Aventis Deutschland GmbH, 65926 Frankfurt am Main, Germany; 3 Department of Clinical Neuroscience, Section of Clinical Neurophysiology, Karolinska Institute, S-141 86 Stockholm, Sweden; 4 Department of Genetics, Institute of Life Sciences and Center for Research on Pain, The Hebrew University of Jerusalem, Jerusalem 91904, Israel; 5 Department of Cell & Animal Biology, Institute of Life Sciences and Center for Research on Pain, The Hebrew University of Jerusalem, Jerusalem 91904, Israel

## Abstract

**Background:**

Nerve injury-triggered hyperexcitability in primary sensory neurons is considered a major source of chronic neuropathic pain. The hyperexcitability, in turn, is thought to be related to transcriptional switching in afferent cell somata. Analysis using expression microarrays has revealed that many genes are regulated in the dorsal root ganglion (DRG) following axotomy. But which contribute to pain phenotype versus other nerve injury-evoked processes such as nerve regeneration? Using the L5 spinal nerve ligation model of neuropathy we examined ***differential ***changes in gene expression in the L5 (and L4) DRGs in five mouse strains with contrasting susceptibility to neuropathic pain. We sought genes for which the degree of regulation correlates with strain-specific pain phenotype.

**Results:**

In an initial experiment six candidate genes previously identified as important in pain physiology were selected for in situ hybridization to DRG sections. Among these, regulation of the Na^+ ^channel α subunit *Scn11a *correlated with levels of spontaneous pain behavior, and regulation of the cool receptor *Trpm8 *correlated with heat hypersensibility. In a larger scale experiment, mRNA extracted from individual mouse DRGs was processed on Affymetrix whole-genome expression microarrays. Overall, 2552 ± 477 transcripts were significantly regulated in the axotomized L5DRG 3 days postoperatively. However, in only a small fraction of these was the degree of regulation correlated with pain behavior across strains. Very few genes in the "uninjured" L4DRG showed altered expression (24 ± 28).

**Conclusion:**

Correlational analysis based on in situ hybridization provided evidence that differential regulation of *Scn11a *and *Trpm8 *contributes to across-strain variability in pain phenotype. This does not, of course, constitute evidence that the others are unrelated to pain. Correlational analysis based on microarray data yielded a larger "look-up table" of genes whose regulation likely contributes to pain variability. While this list is enriched in genes of potential importance for pain physiology, and is relatively free of the bias inherent in the candidate gene approach, additional steps are required to clarify which transcripts on the list are in fact of functional importance.

## Background

Chronic neuropathic pain is a frequent and sometimes devastating sequel of nerve injury. A major cause of this type of pain is axotomy-triggered alteration in the expression of genes in corresponding dorsal root ganglion (DRG) neurons. Gene regulation directly alters the phenotype of neurons that have been axotomized, and in addition may affect neighboring uninjured DRG neurons as well as spinal cord neurons, and glia in both the cord and DRGs. One consequence is the development of electrical hyperexcitability and consequent ectopic neural discharge in affected neurons. Another is altered levels of neurotransmitters, receptors and neuro-immune mediators. These changes can lead to pain hypersensibility through abnormal signal processing in the peripheral and central nervous system [1].

Identification of the genes involved in pain hypersensibility has been the objective of a number of prior studies. This work has revealed that many hundreds of genes are significantly regulated in DRGs in neuropathic pain models (e.g. [2-11]). Only a small fraction of these, however, is likely to contribute to neuronal hyperexcitability and pain. Most are presumably involved in other nerve injury-triggered phenomena such as inflammation, tissue repair, nerve regeneration and apoptosis. Strategies are needed for vetting which changes are causally related to neuropathic pain, and which are not [12]. Here we have implemented such a strategy.

Our approach, correlational analysis, is based on the fact that pain phenotype is highly variable among individuals [13,14]. Correlational analysis begins with quantification of pain phenotype on the one hand, and associated change in gene expression (or any other neurobiological parameter) on the other, in a variety of mouse strains. Then one searches for genes in which the degree of regulation correlates with the degree of altered pain phenotype. If up- or down-regulation of a particular gene transcript is an important pain substrate, then the degree of regulation should correlate across strains with the intensity of the pain.

It is not expected that correlational analysis, alone, will identify individual pain genes. Correlations can arise by chance, particularly considering practical limits on the number of mouse strains that can be analyzed, and the large numbers of transcripts that need to be examined simultaneously. Nonetheless, this approach can deliver an enriched pool of candidates for secondary analysis in mice, and ultimately for confirmation in human pain cohorts. Here we correlated neuropathic pain phenotype with nerve injury-evoked changes in gene expression in DRGs in five inbred mouse strains. First we report a limited test of the idea in which a small number of candidate genes previously implicated in neuropathic pain was hybridized in situ to DRG sections from the five mouse strains. The degree of regulation found was correlated against pain behavior. We then report results of a genome-wide correlational analysis based on regulation data obtained from Affymetrix expression microarrays supported by TaqMan.

## Results

### Strain differences in pain response

Consistent with previous studies using the spinal nerve ligation (SNL) model of neuropathic pain [7,13] we found that tactile allodynia was present in some animals as early as 1 day postoperatively (dpo) and reached a peak by 3 dpo. This peak was maintained until at least 7 dpo, and thereafter it tended to decline. The response magnitude varied among strains (Table 1). The ranking of the three strains tested at 2 dpo, from most to least sensitive, was AKR>B6>CBA (Table 2). This is in agreement with rankings in independent samples of all five strains mice assessed at 4 and 7 dpo (AKR>C58>C3H>B6>CBA, Table 1) although, as is to be expected, the absolute values for hypersensitivity differed somewhat. The ranking for heat allodynia was AKR>CBA>C3H>B6>C58 (Table 1). Autotomy behavior in mice develops at a slower pace than tactile and heat allodynia. It emerges over the first two weeks following hindlimb denervation and then approaches an asymptote [15]. Rank order was C3H>CBA>B6>AKR>C58 (Table 1). RM-ANOVA indicated overall phenotypic differences among the five strains (p < 0.01). It is well established that rank order of response across strains may differ for different nociceptive endpoints [13,14].

**Table 1:** Strain-specific pain phenotype

Mouse strain	Neuroma model *	Tactile allodynia **	Heat allodynia ***
AKR	0.2	58.3	46.1

C57 = B6	1.2	29.8	6.7

C58	0.1	51.3	-20.1

CBA	9.2	5.8	42.9

C3H	9.3	34.5	29.1

* autotomy behavior scored 35 dpo (data from Minert et al. [15]).

** percent reduction in force threshold comparing average preoperative scores to average scores 4 and 7 dpo (data from Mogil et al. [13]; PNI_vf_).

*** percent reduction in response latency comparing average preoperative scores to average scores 4 and 7 dpo (data from Mogil et al. [13]; PNI_ht_).

**Table 2:** Tactile allodynia in three mouse strains as evaluated in this study (S-A).

Mouse strain – surgery	N mice *	Preoperative g ± SD	2 dpo g ± SD	% change ± SD ** (pre-post/pre)
AKR – sham	5	4.59 ± 0.24	4.56 ± 0.24	0.6 ± 4.3%

AKR – SNL	9	4.59 ± 0.21	2.93 ± 0.63	35.8 ± 14.8%

B6 – sham	6	4.89 ± 0.12	4.70 ± 0.18	4.0 ± 3.2%

B6 – SNL	8	4.76 ± 0.14	3.28 ± 0.30	30.7 ± 6.6%

CBA – sham	7	4.78 ± 0.18	4.51 ± 0.26	5.5 ± 5.4%

CBA – SNL	9	4.77 ± 0.23	3.82 ± 0.65	19.8 ± 13.7%

* Number of mice (N), after exclusion of data from 3 mice in the strains that had outlier values in the TaqMan "step 2" analysis, and 7 mice from which too little cRNA was obtained (<12 μg) to permit hybridization on microarrays.

** % change was calculated for each mouse, with results then averaged. Some divergence is present due to rounding.

The analysis of regulation of gene expression using in situ hybridization and microarrays was carried out 3dpo on the grounds that tactile allodynia is not yet fully developed 1 dpo, but is already at a peak by 3 dpo. Thus any gene regulation essential for the initiation and maturation of this pain behavior would already have occurred by 3 dpo. We know that regulation is detectable in many more genes 3 dpo than 1 dpo (see below) and it is likely that the expression of additional and/or different genes might be regulated at later time points. Regulation of such late responding genes might be important for the long-term maintenance of neuropathic pain behavior, or for its eventual decline, but not for its early stages. Such genes would not have been identified in our analysis.

### In situ hybridization

The six genes selected for correlational analysis using in situ hybridization as a monitor of gene regulation were: *Scn10a*, *Scn11a*, *P2rx3, Trpa1*, *Trpv1, Trpm8*. The first two code for voltage sensitive Na^+ ^channel α subunits, the next codes for a purinergic receptor, and the last three code for nociceptive transducer proteins. All six have been strongly implicated in the physiology of pain processing based on studies of regulation in neuropathic pain models and functional association with neuropathic pain phenotypes. [1-11]. As such they are candidates for genes whose regulation might contribute to phenotypic variability among individuals and mouse strains. We stress that this choice of six was not based on prior knowledge of their role in pain variability, and it was by no means unique. Other candidates might just as well have been chosen. The generation of new knowledge concerning the genetics of pain variability was a key objective of the microarray-based correlational analysis.

**Figure 1:**
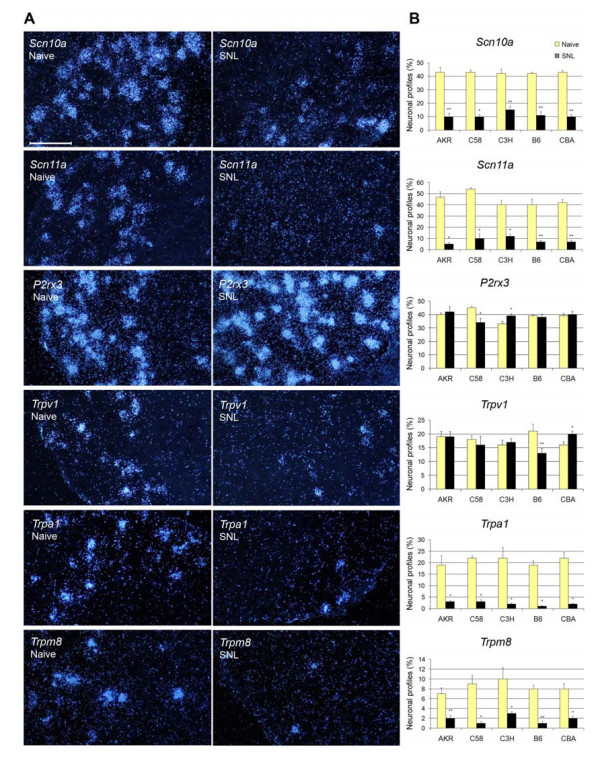
**L5 spinal nerve ligation (SNL) alters the expression of mRNAs in the ipsilateral L5DRG**. **A**, Dark-field micrographs display hybridization signals for each of the six transcripts studied: *Scn10a*, *Scn11a*, *P2rx3*, *Trpv1*, *Trpa1 *and *Trpm8*. DRG sections originate from naïve B6 mice (left column) and B6 mice 3 days after SNL surgery (right column). The scale bar refers to all images: 100 μm. **B**, Column heights (mean ± SEM, n = 4–6) indicate the proportion of DRG neuronal profiles that were positive for expression of each of the six mRNA types in each of the five strains examined. White columns refer to naïve mice; black columns refer to SNL operated mice. Statistical differences between naive and nerve injured mice are indicated with asterisks (Mann-Whitney U test) * p < 0.05; ** p < 0.01.

Each of the targeted genes gave a clear and distinct hybridization signal over DRG neuronal cell bodies. Axotomy induced marked down-regulation of *Scn10a*, *Scn11a, Trpa1 and Trpm8 *in all 5 strains with lesser and variable change observed for *P2rx3 *and *Trpv1 *(Fig. 1).

***Scn10a***, the gene for the TTX-R Na^+ ^channel Nav1.8, was expressed mostly in neuronal profiles of small and medium size in naïve mice. There were no differences in expression at baseline among the five mouse strains (one-way ANOVA, p > 0.05). SNL surgery induced down-regulation in the L5DRG in all strains (Fig. 1; p < 0.05) with no marked change in the size distribution of *Scn10a *-expressing neuronal profiles. The degree of down-regulation of *Scn10a *was not identical across strains, but the across strains correlation was not significant for any of the three pain parameters considered.

***Scn11a***, the gene for the TTX-R Na^+ ^channel Nav1.9, was predominantly expressed in small and medium sized neuronal profiles in naïve mice, with only a minor percentage (highest in the CBA strain) in larger neurons. There was some baseline variation across strains, with higher levels of expression in AKR and C58 mice than in the other strains (Fig. 1). Expression levels were reduced in all strains. Overall, there was a significant across strains difference in the magnitude of down-regulation (p < 0.05), with AKR showing the greatest overall effect and C3H showing the least (Fig. 1b). This yielded a significant negative correlation with spontaneous pain behavior in the neuroma model (r_s _= -0.90, p = 0.04). That is, the less the degree of down-regulation of *Scn11a*, the greater the degree of pain behavior. Correlation with the other pain phenotypes was not significant.

***P2rx3***:, a gene coding one of the P2 purinergic receptors, was almost exclusively expressed in small and medium sized neuronal profiles in naïve and operated mice with some variation among the strains in the percentage of neurons *P2rx3*-positive. Nerve injury altered the level of expression in L5DRGs in a strain dependent manner (p < 0.01). In AKR, B6 and CBA mice it remained unchanged, but in C3H mice it was up-regulated (p < 0.05) while in C58 mice it was down-regulated (p < 0.05, Fig. 1b). The regulation of *P2rx3 *did not correlate significantly across strains with any of the pain phenotypes considered.

***Trpv1***, which codes for the capsaicin receptor, was mostly expressed in small neuronal profiles of all five strains. Following nerve injury there was a shift towards overall larger *Trpv1*-positive neuronal profiles in CBA mice but not in any of the other strains. The proportion of neuronal profiles labeled with *Trpv1 *was significantly increased in the CBA mice (p < 0.05), but it was decreased in B6 mice (p < 0.01, Fig. 1b). Overall, the regulation of *Trpv1 *did not correlate significantly with any of the pain parameters considered.

***Trpa1***, which codes for a receptor for noxious cold and a variety of irritating chemicals, was expressed almost exclusively in small and medium sized neuronal profiles in naïve mice. Spinal nerve injury did not cause a major change the sizes of *Trpa1*-positive neuronal profiles in the L5DRG. However, it caused dramatic down-regulation, to almost undetectable levels, in all five strains (Fig. 1). Overall, the regulation of *Trpa1 *did not correlate significantly with any of the pain parameters considered. For *Trpa1 *(and *Trpm8*) we also examined gene expression in the L4DRG. L5 spinal nerve injury did not induce any detectable change in expression in this ganglion despite the pronounced effect in the L5DRG.

***Trpm8***, the gene for a cold and menthol receptor, was mostly expressed in small neuronal profiles in naive and operated animals. Like *Trpa1, Trpm8 *was strongly down-regulated in the L5DRG in all strains at 3 dpo, with expression nearly abolished in some strains (Fig. 1). A significant negative across strains correlation was seen between the degree of down-regulation of *Trpm8 *and heat allodynia (r_S _= -0.98, p = 0.005; r_P _= -0.90, p = 0.04). The greater the down-regulation of *Trpm8 *the less the heat allodynia. Correlation with the other pain phenotypes was not significant. L5 spinal nerve injury did not change the level of *Trpm8 *mRNA expression in the L4DRG.

### TaqMan analysis

Taqman analysis was first applied to a small number of gene transcripts in "step 1" pilot experiments using B6 mice. There were two objectives: 1) to facilitate development of the methods needed for microarray analysis based on the small amounts of mRNA extractable from individual mouse DRGs, and 2) to evaluate the sensitivity of the system using marker genes predicted, on the basis of prior studies, to be regulated in the L5DRG following L5 spinal nerve transection. Expression in ganglia taken from mice 3 dpo (n = 10) was compared to the corresponding ganglia from sham operated mice (n = 8). Eight genes were examined including two drawn from the in situ hybridization analysis. Seven of the eight are known to be significantly regulated in L5DRGs in rats following sciatic nerve transection (*Gal, Pacap, Cchl2a, Sprr1a, C-Jun, Trpv1 *and *Scn11a*) while one is not regulated (*Mmpcp1; *[2]).

**Figure 2:**
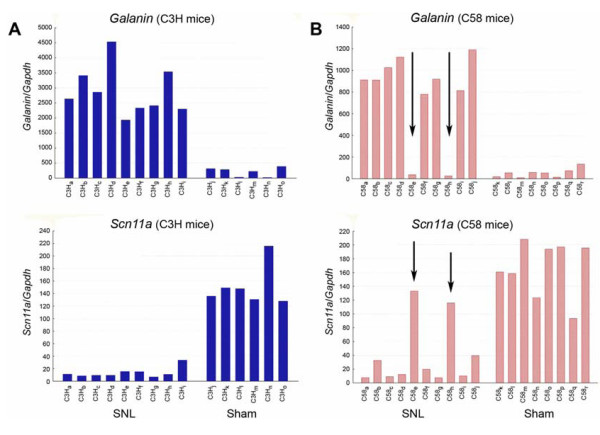
***Galanin *is up-regulated and *Scn11a *is down-regulated following spinal nerve ligation (SNL)**. Gene expression, plotted in relation to that of the stable housekeeping gene *Gapdh*, was assessed using TaqMan analysis 3 days following L5 SNL. Expression profiles for both genes are shown for individual mice of the **A**, C3H strain (labelled C3H_a-o_) and **B**, C58 strain (labelled C58_a-r_). Arrows in **B**, indicate two outlier mice in which expression levels of both *Galanin *and *Scn11a *correspond to sham operated animals, suggesting that spinal nerve injury had not been carried out adequately.

Reproducibility among replicate samples from individual DRGs was high as reflected in very low coefficients of variation, as was consistency across mice in the operated and sham groups (Fig. 2). TaqMan analysis revealed that the seven genes regulated in rat DRGs are likewise regulated in the mouse, whereas *Mmpcp1*, which is not regulated in the rat, is also not regulated in the mouse.

On the basis of these results one of the up-regulated genes (*Gal*, which codes for the neuropeptide galanin) and one of the down-regulated genes (*Scn11a*) were chosen as marker genes for "step 2" TaqMan analysis. Here we compared baseline gene expression, and regulation following SNL surgery, in all five mouse strains. As observed for B6 mice in "step 1", individual variability was low within strains, confirming the feasibility of carrying out across strains comparisons. Results for two strains, C3H and C58, are shown in figure 2. The degree of resolution among DRGs from individual mice was sufficient to allow us to use these data to independently verify that the SNL surgery and the DRG identification had been done as intended, before actually applying cDNA to the expression microarrays. This facility is illustrated by outlier values for *Gal *and *Scn11a *expression in two ostensibly nerve injured mice, both C58. Expression levels in these two mice corresponded to sham operated mice and differed significantly from the other nerve injured mice (arrows in Fig. 2). Importantly, the outlier data came from the same individual mice in the case of both marker genes. Our interpretation is that in these two mice either the L5 segment had been misidentified during surgery or tissue dissection, or that the nerve injury had been incomplete. All data from these two outlier mice were removed prior to microarray analysis. No such outliers occurred among the C3H or B6 mice. Outlier values for *Gal *and *Scn11a *were also found in two AKR mice (one operated, one sham) and in one operated CBA mouse. These animals were also eliminated from the microarray analysis.

### Microarray analysis of gene expression and regulation

Profiles of gene expression were generated using Affymetrix mouse whole-genome microarrays for the L5 and L4 DRGs on the operated side for each individual mouse in all five strains. Nerve-injury induced regulation was then calculated using the Resolver software by comparing like ganglia taken from individual nerve injured mice and the average of sham operated mice as described in the Methods. This yielded a value for fold up- or down-regulation for each gene, for both ganglia. For the strains-based analysis values for the individual ganglia were averaged within strains to yield the mean fold change (± variance) of each expressed gene for each of the five strains. Substantial differences were observed across strains in the baseline expression level of many genes as determined from expression profiles in the sham operated animals. Likewise, and most important for the present analysis, there were substantial across-strains differences in the degree of gene regulation comparing nerve injured and sham operated mice of a given strain. For some of these genes, strain-specific differences in regulation correlated with strain-specific difference in nerve injury-induced pain behavior.

In all five strains a large number of transcripts met our predetermined criterion for significant regulation as shown in Table 3. Averaging over the five mouse strains a mean of 2,552 ± 477 genes were significantly regulated in the L5DRG, representing 11.6% of the 22,002 genes significantly expressed, on average. The 20 genes with the greatest fold up-regulation, and the 20 with the greatest fold down-regulation, in one of the strains (AKR, 3dpo) are provided as on-line supplemental material (see additional file 1). The number of genes regulated had doubled from 1 dpo to 3 dpo in the two strains studied at both time-points (Table 3). At 3 dpo this number ranged from 1931 (in B6 mice) to 3053 (in C58 mice), with 3927 transcripts significantly regulated in at least one strain. There was only a small preponderance of down-regulated genes over up-regulated genes. Results obtained in the TaqMan analysis of *Gal *and *Scn11a *transcripts measured in the L5DRGs of all five strains were confirmed in the microarrays.

**Table 3:** Number of gene transcripts significantly expressed, and significantly regulated, following transection of the ipsilateral L5 spinal nerve.

**Mouse strain**	dpo*	**L5DRG**				**L4DRG**			
		
		**significantly expressed**	**significantly regulated**			**significantly expressed**	**significantly regulated**		
		total	total	up- regulated	down- regulated	total	total	up- regulated	down- regulated

**C3H**	1	21936	1357	753	604	-	-	-	-

**C3H**	3	20526	2404	1132	1272	22436	15	9	6

**C58**	1	21507	1281	650	631	-	-	-	-

**C58**	3	19750	3053	1477	1576	22888	72	60	12

**AKR**	3	22738	3014	1477	1537	23238	20	18	2

**B6**	3	23021	1931	871	1060	21866	0	0	0

**CBA**	3	23978	2356	1014	1342	22345	13	7	6

**mean ± SD** **n = 2 C3H, C58**	1	21722	1319	702	618	-	-	-	-

**mean ± SD** **n = 2 C3H, C58**	3	20138	2729	1305	1424	22662	44	35	9

**mean ± SD** **n = 3 AKR, B6, CBA**	3	23246 ± 650	2434 ± 546	1121 ± 317	1313 ± 240	22483 ± 696	11 ± 10	8 ± 9	3 ± 3

**mean ± SD** **n = 5 all strains**	3	22003 ± 1784	2552 ± 477	1194 ± 274	1358 ± 210	22555 ± 527	24 ± 28	19 ± 24	5 ± 5

* DRGs were harvested either 1 or 3 days postoperative (dpo)

The number of genes significantly regulated in the L4DRG (not axotomized, "uninjured") was much lower than in the L5DRG, averaging only 24 ± 28 across strains (p < 0.0001, range: 0–72, Table 3). These accounted for only 0.1% of the 22,555 genes significantly expressed in the L4DRG. There was no overlap in these regulated genes among strains. In total only 120 transcripts were regulated in the L4DRG in one strain or more.

### Correlational analysis (microarrays)

Correlational analysis was carried out in two alternative ways: 1) based on average regulation (fold-change) data for separate cohorts of the five mouse strains (Table 1), and 2) based on regulation data vs. tactile allodynia values derived from the same individual mice, in the three strains in which individual behavioral data were collected (Table 2). Expression data were plotted against behavioral data, and both Pearson (parametric) and Spearman (rank) coefficients of correlation were calculated (r_p_, r_s_). For the strains-based analysis plots were made independently based on all three pain phenotypes, tactile and heat allodynia in the SNL model and autotomy behavior in the neuroma model. For the individual mouse-based analysis, tactile allodynia was the only behavioral parameter available.

#### Five strains analysis

Overall, when correlated against strain specific measures of tactile allodynia in the SNL model of neuropathic pain (Table 1), 144 transcripts yielded an (uncorrected) p-value of ≤ 0.05 (which corresponds to r_P _>0.878 assuming normal distribution of r_P_). These 144 transcripts represent 5.6% of the 2552 transcripts that, on average, met the filtering criteria for significant regulation in the L5DRG of the five mouse strains. The top transcripts among these, ranked by r_P_, are given in Table 4, along with the corresponding values of r_S_, corresponding p-values and the q-value derived from the FDR. When correlated against strain specific measures of thermal allodynia (Table 1) r_p _for 141 (5.5%) of the regulated genes had a p-value of 0.05. When correlated against spontaneous pain behavior (autotomy, Table 1) in the neuroma model r_p _for 45 (1.8%) of the regulated genes had a p-value of ≤ 0.05.

**Table 4:** Correlational analysis of log ratio data from the L5DRG (nerve injured vs. sham) against across-strain measures of tactile allodynia (five strains analysis).

**Affymetrix qualifier**	Gene name *	**Proposed gene function**	**r**_**P**_	**p-value of r**_**P**_	**r**_**S**_	**p-value of r**_**S**_	**q-value from FDR**
1416827_at	Tbxas1	Thromboxane synthase	0.998	<0.001	1.000	<0.001	0.141

1450792_at	Tyrobp	Protein tyrosine kinase binding protein	0.997	<0.001	1.000	<0.001	0.217

1416001_a_at	Cotl1	Regulation of actin cytoskeleton	0.996	<0.001	1.000	<0.001	0.217

1427076_at	Mpeg1	Macrophage expressed gene	0.995	<0.001	0.900	0.037	0.241

1419483_at	C3ar1	Complement component receptor	0.994	0.001	1.000	<0.001	0.259

1423547_at	Lyzs	Lysozyme	0.993	0.001	1.000	<0.001	0.259

1438320_s_at	Mcm7	DNA replication licensing complex	0.993	0.001	0.900	0.037	0.259

1421477_at	Cplx2	Complexin 2	-0.993	0.001	-1.000	<0.001	0.259

1425108_a_at	BC004728	EST (expressed sequence tag)	0.992	0.001	1.000	<0.001	0.259

1437829_s_at	Eef2k	Transcribed locus	0.988	0.002	1.000	<0.001	0.424

1450842_a_at	Cenpa	Cell cycle regulator	0.988	0.002	1.000	<0.001	0.424

1427368_x_at	Fes	Feline sarcoma oncogene	0.986	0.002	1.000	<0.001	0.489

1436778_at	Cybb	Cytochrome, beta polypeptide	0.985	0.002	1.000	<0.001	0.490

1419943_s_at	Ccnb1	Mouse cyclin B1	0.984	0.002	1.000	<0.001	0.517

1424829_at	A830007P12Rik	EST	0.984	0.003	0.900	0.037	0.517

1416612_at	Cyp1b1	Cytochrome involved in angiogenesis	0.983	0.003	0.900	0.037	0.518

* Transcripts with Pearson correlation coefficient (r_P_) among the highest observed are listed, along with their corresponding p-values, Spearman rank correlation coefficient (r_S_) and p-values, and q-value from FDR analysis.

There was little overlap in the lists of correlated genes among the three pain phenotypes examined. There were only three correlated transcripts in common between tactile allodynia and autotomy (Rassf4, Olfm2, genename), and one between tactile allodynia and heat allodynia (D430015B01Rik). No correlated transcripts were shared by heat allodynia and autotomy (nor any among all three). As noted, due to massive multiple testing, no correlation coefficient reached statistical significance after Bonferroni correction. In the FDR analysis only one gene, *Pde6b*, which codes for a phosphodiesterase, obtained a significant q-value (q = 0.005, in relation to autotomy behavior). Functional analysis based on gene ontology categories (GOTM and IPA) showed that the pain-correlated genes fell into large categories roughly in proportion to their representation in the whole mouse genome, but with significant enrichment in categories related to immune and inflammatory processes and the regulation of cellular metabolism, especially for genes correlated to tactile allodynia.

In the L4DRG only 24 transcripts, on average, showed significant regulation following L5 spinal nerve injury (120 transcripts in at least one strain; Table 3). None of these achieved a q-value of ≤ 0.05 when correlated against tactile or heat allodynia, although one reached this criterion when correlated against autotomy behavior (*Mcm10*, q = 0.027).

#### Analysis based on data from individual mice (three strains)

The second mode of correlational analysis was based on gene expression and behavioral phenotype (tactile allodynia only) from the individually phenotyped AKR, CBA and B6 mice. For each significantly regulated gene we plotted the degree of tactile allodynia in each mouse (see Methods) against the log-ratio of regulation of the gene expression signal in that mouse's L5DRG. Regulation was based on a comparison with mean expression values from the sham operated mice of the same strain. In addition we plotted the degree of allodynia against the absolute signal intensity for gene expression in the axotomized L5DRGs. Both plots included 26 data points (9 AKR mice, 9 CBA mice and 8 B6 mice).

**Figure 3:**
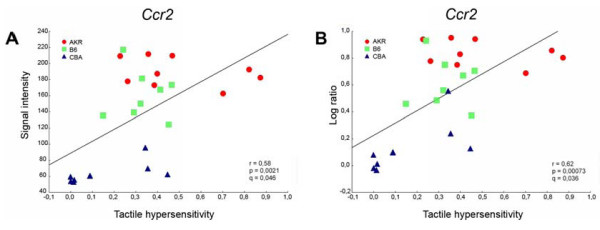
**Regulation of expression of the cytokine receptor *Ccr2 *after L5 SNL correlated positively with the degree of tactile hypersensitivity**. Each symbol represents one mouse of the AKR, CBA or B6 strain. Expression data, for individual mice, are plotted as: **A**, the raw signal intensity values associated with *Ccr2 *gene expression, and **B**, the log ratio of the signal intensity of the individual mouse divided by the average signal intensity of all sham operated mice of the same strain. Values for tactile hypersensitivity of the individual mice were normalized as indicated in the Methods, where increasing values indicate greater sensitivity. r = Pearson correlation coefficient, p = statistical significance of r, q =false discovery rate (FDR) coefficient associated with *Ccr2 *in the microarray analysis.

**Figure 4:**
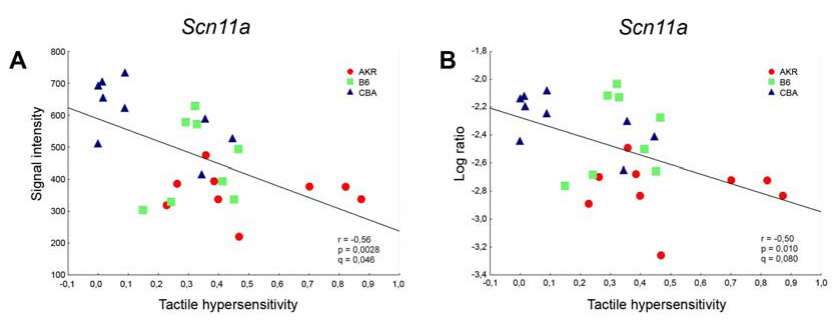
**Regulation of expression of the TTX-R Na^+ ^channel alpha subunit *Scn11a *after L5 SNL correlated negatively with the degree of tactile hypersensitivity**. Each symbol represents one mouse of the AKR, CBA or B6 strain. Expression data, for individual mice, are plotted as: **A**, the raw signal intensity values associated with *Scn11a *gene expression, and **B**, the log ratio of the signal intensity of the individual mouse divided by the average signal intensity of all sham operated mice of the same strain. The value for tactile hypersensitivity for these mice was normalized as indicated in the Methods, where increasing values indicate greater sensitivity. r = Pearson correlation coefficient, p = statistical significance of r, q = false discovery rate (FDR) coefficient associated with *Scn11a *in the microarray analysis.

Based on the log-ratio data 108 out of the 2434 transcripts (4.4%) that were significantly regulated on average in these three strains yielded a q-value of ≤ 0.05 based on log ratio expression values (Table 3). From measurements of absolute signal intensity 203 of the 2434 transcripts (8.3%) met this criterion. Overlap in these two lists was considerable. Nearly half of the transcripts in the list of 108 transcripts also appeared on the list of 203 transcripts (46/108). Twenty seven of the transcripts in the list of 108 were also among the 144 transcripts obtained from the five strains analysis of transcripts correlated to tactile allodynia. Data for two of the 108 transcripts, the chemokine receptor *Ccr2 *and the TTX-R Na^+ ^channel gene *Scn11a*, are shown in figures 3 and 4. Both of these genes have been previously implicated in neuropathic pain processing based on other approaches [16-18]. Breakdown into functional categories (GOTM and IPA) was similar to that obtained in the five strains analysis for transcripts correlated to tactile allodynia.

For the six genes studied using in situ hybridization there was a good correspondence in the overall pattern of gene regulation in the L5DRG as determined by the in situ and the microarray (and the TaqMan) analyses. That is, all of the methods showed marked down-regulation of *Scn10a*, *Scn11a, Trpa1 and Trpm8 *with lesser and variable change in *P2rx3 *and *Trpv1*. Across strain rankings of regulation, however, were not always the same. For two of the genes rankings of the degree of regulation based on in situ hybridization and microarray analysis had a significant positive correlation (*Scn10a *(r_s _= 0.89, p = 0.04) and *Trpv1 *(r_s _= 0.87, p = 0.05)). However, the remaining four did not.

In the L4DRG three genes, *Bdnf, Steap1 *and *ApoH *(which code for the neurotrophin BDNF, a prostate epithelial transmembrane antigen, and apolipoprotein H, respectively) showed a significant q-value for correlations based on log ratio data, but none did so for correlations based on absolute signal intensity. The three correlated genes from the L4DRG were not among the 108 correlated genes from the L5DRG.

## Discussion

Primary sensory neurons in DRGs undergo a massive change in gene expression in experimental models of neuropathic pain, changes that undoubtedly contribute to altered somatosensory signal processing in the event of neuropathy [2-11]. In our material, >11% of all expressed genes, 2552 on average, were found to be significantly up- or down-regulated in the L5DRG 3 days following transection of the L5 spinal nerve. This number is larger than prior estimates probably because of the proximal location of the lesion, and the improved protocol for detecting change. In common with prior estimates, however, it highlights a fundamental challenge. The sheer number of regulated genes means that additional methods are needed to identify which individual transcripts play an important role in pain sensation versus other processes induced by axotomy [12]. Using correlational analysis we considerably shortened the list of candidates. In contrast, we found that very few genes were regulated in the L4DRG following transection of the L5 spinal nerve. This is surprising in light of functional studies that have argued that abnormal activity in "uninjured" L4DRG nociceptors may be important for the development of tactile allodynia in the SNL model [19]. Perhaps the relevant changes occur in sensory signaling processes not related to altered gene expression in the L4DRG. The observations on the L4DRG confirm that the massive regulation seen in the L5DRG was in fact related to axotomy.

We recognize that the shortened gene list still includes many transcripts unrelated to neuropathic pain. Assuming random assortment, 5% of transcripts are expected to correlate with pain phenotype at p ≤ 0.05 by chance alone. However, despite residual contamination transcripts whose regulation is functionally related to pain behavior are expected to be retained at higher frequency than pain-neutral transcripts, yielding significant enrichment. At the most optimistic, assuming that all functionally relevant genes were captured by our correlation procedure, at least 95% enrichment is expected. Thus, while the lists of correlated transcripts remain too long to permit direct selection of candidate pain genes, they constitute a valuable and relatively bias-free look-up table, a tool for screening candidates derived from other approaches. The list, however, is clearly not complete. In addition to pain-correlated genes missed, there is no inherent biological contradiction to the possibility that some genes are functionally related to pain phenotype but that their regulation does not contribute importantly to strain differences.

Might the gene lists be further enriched by increasing the stringency of the selection criterion from p ≤ 0.05 to, say, p ≤ 0.01? While not impossible, given the intrinsic noise present in the phenotyping process we believe that this step would be more likely to eliminate functionally significant transcripts. This is because within the enriched list, genes with the highest correlation coefficients are not much more likely to be functionally related to pain than those with lesser, but still high correlation coefficients. Further vetting requires additional biological information. For example, there is a clear benefit to increasing the number of data points used to generate correlations. We accomplished this in three of the five strains studied by collecting phenotypic data (on tactile allodynia) from individual mice rather than relying on strain means. This resulted in a reduction in the number of genes with significant correlation coefficients, presumably by removing more false positive results than true positive results. Related approaches are to increase the number of mouse strains studied and the behavioral diversity among them.

Two other enrichment approaches have been attempted in the past. In one, lists of regulated transcripts were assembled using a variety of different rodent models of painful neuropathy [11,20,21]. As expected, for each model hundreds of transcripts were significantly regulated in the axotomized DRGs. Lists were shortened by identifying genes similarly regulated in more than one model. This strategy is likely to reduce random noise, but it is also likely to capture regulated genes that are not related to pain. Transcripts related to regeneration and apoptosis, for example, would all be positively selected for. The approach also makes the risky assumption that different pain models (diagnoses) share the same underlying pathophysiology. If this is not the case then pain-related genes would be systematically excluded.

A second approach compared genes regulated in the DRG following nerve injury in two rat strains that had a consistent difference in pain phenotype in the SNL neuropathy model [4]. Although closer in concept to our study, the rat strains used were not congenic, or even particularly close in genetic background. For this reason observed differences in gene regulation may well have reflected pain-neutral differences in genetic background. Correlations based on only two strains provide zero degrees of freedom. We used five strains, and alternatively 26 individual mice (from three strains) to generate correlations. In this context it is worth noting that not only does baseline gene expression level within a particular tissue (e.g. DRG) vary among mouse strains, but it also varies among tissues and among cell types within tissues [22,23]. Our in situ hybridization data affirm this to be the case with respect to DRG neurons of varying size, at least for some transcripts. An example is up-regulation of *Trpv1 *in CBA mice and down-regulation in B6 mice, and the de novo appearance of *Trpv1 *in DRG cells of medium and large size, but only in CBA mice.

An intrinsic limitation of microarray based studies, including ours, is that massive multiple hypothesis testing undermines the ability to establish statistical significance for any given transcript identified. This limitation is only partly eased using FDR analysis [24]. Ultimately, our enriched list of pain-related genes needs to be subjected to secondary screens. In situ hybridization is one example. Although this method is too resource intensive to be used to screen large numbers of transcripts, it represents an independent implementation of correlational analysis. The overall pattern of gene regulation seen in the arrays was reiterated in all six candidate genes subjected to in situ analysis. However, the across-strains pattern was reproduced in only two of the six. There are several possible reasons for this difference between microarray and in situ measurements. Microarray (and TaqMan) analysis integrates over the entire ganglion, including neurons, glia and other resident cells, and includes cells with low expression levels. The cellular source of the mRNA is not identified. Likewise, the method does not take into account the possibility of up-regulation in one cell population balanced by down-regulation in another. Finally, stable expression levels in many cells may mask significant regulation in a small but important subpopulation.

Interestingly, the degree of regulation correlated significantly with pain phenotype for two of the six candidate genes studied with in situ hybridization (33%;*Scn11a *and *Trpm8*). This yield was much higher than for the array analysis which considered all regulated genes (p < 0.001). This outcome, which presumably reflects the additional information that underlay the choice of the six candidates, lends validation to the correlational approach. Regulation of the Na^+ ^channel α subunit *Scn11a *correlated with levels of spontaneous pain behavior, and regulation of the cool receptor *Trpm8 *correlated with heat hypersensibility. For *Scn11a*, expression was reduced in all strains, with the degree of reduction minimal in animals that exhibited a high level of ongoing pain behavior and maximal in strains with minimal ongoing pain. A good deal of evidence links Na^+ ^channels, including *Scn11a*, to the emergence of ectopic afferent hyperexcitability after nerve injury and consequent spontaneous firing and spontaneous neuropathic pain [1,16,25]. The observed correlation is therefore consistent with pain-protected mouse strains showing the greatest level of *Scn11a *down-regulation. We note, however, that mice with null mutation of *Scn11a *continue to show tactile allodynia in neuropathy models [26,27]. These knockout mice have not been checked for autotomy.

For *Trpm8*, down-regulation occasioned reduced heat allodynia. *Trpm8 *functions as a cold transducer in primary afferent nociceptors [28]. The relation to thermal sensation is obvious, but the link to altered heat response requires further investigation. Cold allodynia in the SNL model has not been compared in our series of mouse strains. In intact mice, however, sensitivity to cold is genetically correlated with sensitivity to heat [29]. We stress that failure to find a significant correlation between gene regulation and pain phenotype in the other four transcripts tested using in situ hybridization does not mean that the corresponding genes, or their regulation following nerve injury, is not important for pain phenotype. Each of these genes may well play an essential role in pain physiology. However, the result indicates that their degree of regulation does not contribute much to across strain variability, at least in the five strains examined.

## Conclusion

Despite impressive progress in recent years it remains challenging to identify genes whose expression contributes to a predefined functional endpoint such as pain behavior. Correlational analysis is a means of facilitating such identification. Correlational analysis of genes already known to be related to pain provided evidence that in the case of *Scn11a *and *Trpm8*, differential regulation following nerve injury plays a role in phenotypic variability across strains. Applied in a large scale format to data obtained from expression microarrays correlational analysis yielded an entire look-up table of genes whose regulation likely contributes to variability in pain phenotype. As such, many of the genes on the list probably play a significant role in the physiology of neuropathic pain. In principle, the approach used can be applied to any relevant source of biological data, and corresponding phenotypes.

## Methods

### Animals

We used young adult males (8–19 weeks of age) of five different inbred mouse strains obtained from The Jackson Laboratory (Bar Harbor, ME, USA). The strain were: AKR/J (AKR), CBA/J (CBA), C3H/HeJ (C3H), C57BL/6J (B6 or C57) and C58/J (C58). Experiments were carried out at two different venues, the Karolinska Institutet (KI, Stockholm) and Sanofi-Aventis Deutschland GmBH (S-A, Frankfurt). Both before and after experimental surgery mice were maintained in groups in transparent plastic cages bedded with wood shavings (Lantmännen, Kimstad, Sweden at KI) or Lignocel HBK 1500/3000 (JRS, Resenberg, Germany at S-A). Water and food pellets (RM3 pellets, Special Diets Services, Witham, UK at KI; product R/M-H 10 mm, Ssniff, Soest, Germany at S-A) were available *ad libitum*. At both venues the light:dark cycle was 12:12 with lights on at 06:00 AM. All procedures were carried out with the approval of the Institutional Animal Care and Use Committee of KI or in accordance the German Animal Protection Law as overseen by the local Ethics Committee for Research on Laboratory Animals at S-A.

Experiments were based on the SNL model of neuropathic pain [30] with slight modifications. For in situ hybridization analysis (KI) we used a total of 40 mice, 8 from each of the five strains. For each strain 6 mice were sacrificed 3 dpo and 2 were intact controls. For TaqMan and microarray analysis (S-A) we used a total of 144 mice. Observations were made at two postoperative time points, 1 dpo for C58 and C3H mice and 3 dpo for all five strains. In each group 10 mice underwent SNL surgery and 8 mice underwent sham surgery. An additional group of 18 B6 mice (10 SNL and 8 sham) were used for "step 1" TaqMan pilot observations (see below). In all five strains measurements of tactile and heat allodynia in the SNL model of neuropathic pain, and of spontaneous pain in the neuroma model of neuropathic pain, were based on our observations on other individuals of these same strains [13,15]. The reason is that we wanted to track pain behavior in the strains beyond 3 dpo. However, in three stains we made behavioral measurements of tactile allodynia 2 dpo on the actual, individual mice that were sacrificed on the following day for use in the microarray analysis.

### Surgical procedures

Mice were anaesthetized with 350 mg/kg i.p. chloral hydrate (KI) or 1.5–3% isoflurane in a 1:2 mixture of O2 and N2O (S-A). Lower back skin was shaved and wiped with 70% ethanol. Using sterilized instruments an incision was made through the skin and the paraspinal muscles were separated from the spinous processes at the L5-L6 levels. The L6 transverse process was then removed exposing the L5 spinal nerve. Finally, the spinal nerve was transected 3–5 mm distal to the ganglion, without ligation, and a 3–4 mm segment was removed from the distal nerve stump. Care was taken throughout to avoid damage to the adjacent L4 and L6 nerves. To obviate the risk of severing the wrong spinal nerve, at terminal dissection the nerve identified as L5 was dissected into the lumbosacral plexus to insure that it indeed made a major contribution to the sciatic trunk. Sham operated control mice underwent the identical surgical exposures but nerves were not transected. In all animals muscle and skin incisions were closed in layers using surgical sutures and stainless steel clips (Stoelting, Wood Dale, Illinois, US). Mice were given buprenorphine as post-operative analgesic (0.06 mg/kg subcu., S-A) and following recovery from the anesthetic they were returned to their home cage.

### Behavioral testing

We obtained data on tactile allodynia, heat allodynia and spontaneous pain in the neuroma model on independent groups of mice of the same five strains used to assess gene regulation. Methods and results were reported previously, with key values presented in Table 1[13,15]. Briefly, SNL surgery was carried out as described above. Tactile allodynia was assessed using von Frey monofilaments and heat allodynia was assessed by measuring withdrawal latency to a light beam projected from below onto the plantar hindpaw. Autotomy, the behavioral endpoint in the neuroma model of spontaneous neuropathic pain [31,32], was scored at weekly intervals for 5 weeks following transection of the sciatic and saphenous nerves. The scoring protocol was according to Wall et al. [31]. Briefly, one point was registered for loss of one or more toe nails and an additional point was scored for injury to the distal and the proximal half of each toe for a total maximal score of 11. For calculation of the Spearman rank correlation statistic (r_s_) behavioral scores were converted to strain ranks (1 to 5) where 1 represents the strain with the least nerve injury-induced behavioral change and 5 represents the strain with the largest change.

In addition, in the AKR, B6 and CBA strains, we obtained behavioral data on tactile allodynia from the individual mice used for the array analysis. Two days after SNL surgery the mice were placed separately in a 10 × 10 cm enclosure. Animals were habituated to the test environment for at least 30 minutes and then cutaneous sensitivity of the lateral plantar aspect of the hind paw was determined using the automated dynamic plantar aesthesiometer (Ugo Basile, Comerio, Italy). In this apparatus a metal filament 0.5 mm in diameter automatically rises from below the wire grid floor and applies a force that increases linearly from 0 to 5 g in 10s. The force that elicited paw withdrawal by the mouse, registered automatically by the apparatus, was taken as the animal's response threshold for a given trial. Each paw was tested four times over a period of 90–120 min with at least 30 min rest between tests. Tactile (hyper) sensitivity was defined as the average of the four measurements. For correlational analysis, the degree of tactile allodynia was calculated as the natural logarithm (ln) of threshold measurements for (SNL contralateral paw/SNL ipsilateral paw) - ln (mean of sham group contralateral paw/mean of sham group ipsilateral paw).

### In situ hybridization

Three days postoperative mice were deeply anaesthetized with chloral hydrate (350 mg/kg i.p.) and perfused transcardially with 20 mL Tyrode's solution. Identification of the L5 spinal nerve and DRG was confirmed by a carbon mark left during surgery. DRGs L4 and L5 were quickly dissected out, snap frozen on dry ice and stored at -70°C for up to 6 days until sectioning. For operated mice DRGs were taken from the side of the SNL injury while for intact controls they were taken from either side. DRGs of a given level from operated and control animals of each strain were embedded side by side. Further, DRG pairs of this sort from all five strains were embedded together in single blocks for simultaneous processing using Tissue-Tek™ OCT compound (Sakura, Zoeterwounde, Netherlands). Blocks, each containing 10 DRGs, were cryo-sectioned at 12 μm and thaw-mounted onto Super Frost/Plus slides (Menzel GmbH, Braunschweig, Germany). This multiple embedding protocol ensured that sets of experimental and control ganglia from each mouse strain were processed in parallel under identical conditions.

Oligonucleotide probes were synthesized complementary to the mouse mRNA sequences of the Na^+ ^channel α-subunit Na_v _1.8 [GenBank: NM_009134], (base 2980–3021), the Na^+ ^channel α-subunit Na_v_1.9 [GenBank: NM_011887], (base 3429–3470), the ATP receptor P2X_3 _[GenBank: NM_145526], (base 1123–1164), the heat- and capsaicin-sensitive receptor TRPV1 [GenBank: NM_001001445], (base 531–572), the noxious cold-sensitive receptor TRPA1 [GenBank: NM_177781], (base 739–780) and the cooling- and menthol-sensitive receptor TRPM8 [GenBank: NM_134252], (base 809–850); Cybergene, Huddinge, Sweden). Procedures for in situ hybridization were as previously described [33], but with slight modifications. Briefly, oligonucleotides were 3'-end labelled with (^33^P)-dATP using terminal deoxyribonucleotidyl transferase (TdT). Sections were hybridized for 16–20 hours at 42°C in a humidified chamber with ~7 μL probe (~1.5 × 10^5 ^cpm/μL) made to approximately 200 μL solution for each slide, in a mixture of 4× SSC (standard saline citrate) buffer (1× SSC = 0.15 M NaCl, 0.015 M sodium citrate), 50% formamide, 1× Denhardt's solution (0.02% each of polyvinyl-pyrrolidone, bovine serum albumin and Ficoll), 1% sarcosyl, 0.02 M phosphate buffer (pH 7.0), 10% dextran sulphate, 500 mg/mL heat denaturated salmon sperm DNA and 200 mM dithiothreitol. Slides were then rinsed 5× 15 minutes at 60°C in 1× SSC and the last rinse was allowed to cool to room temperature. Slides were dipped in distilled water, dehydrated through graded ethanols (70%, 90%, 99.5%), air-dried, dipped in photographic emulsion (Kodak NTB2, diluted 1:1 in distilled water) and exposed at 4°C for 2–4 weeks. The slides were then developed, fixed and coverslipped using Pertex mounting medium (CellPath Plc, Hemel Hempstead, UK).

### Quantification of in situ hybridization

Quantitative analyses of mRNA hybridization signals used a Nikon E600 microscope equipped with a darkfield condenser and a Nikon DXM 1200 digital camera. Images were captured under darkfield and brightfield illumination to facilitate identification of cell borders. Using Easy Image software (EI 3000, v.3000; Tekno Optik AB, Huddinge, Sweden) the darkfield image was thresholded based on pixel intensity. This mask was overlaid on the bright field image. The circumference of neuronal profiles in the field of view was outlined with an on-screen cursor, excluding profiles that had an area < 150 μm^2^. For each neuronal profile outlined, EI3000 extracted area and labelling intensity, which were used for subsequent calculation of the signal/noise (S/N) ratio. S/N ratio was based on comparison of mean pixel intensity within the perimeter of the cell and the background. Additional fields were sampled until a minimum of 150 neuronal profiles were evaluated per animal for each type of mRNA hybridization. Neuronal profiles were categorized by size as follows: small (150 ≤ 300 μm^2^), medium (300 ≤ 600 μm^2^) and large (> 600 μm^2^). Neuronal profiles with S/N ratio ≥ 5 were counted as "labelled" and the proportion of such labelled cells was calculated.

Detailed morphometric data will be given elsewhere. Here we focus on regulation. Change in expression (i.e. "regulation") for each marker examined, in each strain, was the ratio of the proportion of cells labelled comparing operated and control DRGs from matched pairs. All analyses were performed blind.

### RNA extraction for TaqMan and microarray analysis

One or three days after the surgery mice were killed by CO_2 _inhalation and the lumbar spine was divided longitudinally. Identification of the L5 spinal nerve and DRG was confirmed by dissection proximally into the sciatic nerve. L4 and L5 DRGs were removed into PBS, frozen on dry-ice and stored at -70°C. After DRGs from all groups were collected, total RNA was isolated from the individual ganglia using the PicoPure RNA Isolation Kit following the manufacturer's protocol (Arcturus Bioscience Inc, Mountain View, CA, USA). Average RNA yield was ca. 500 ng/DRG. The high quality of the RNA extracted was verified using the Agilent RNA 6000 Pico Kit (Agilent Technologies, Santa Clara, CA, USA).

### cDNA synthesis and TaqMan analysis

cDNA synthesis was performed using the Applera Ltd. (Norwalk, CT, USA) Reverse Transcriptase Kit (N8080234) and RNase Inhibitor (N8080119). cDNA synthesis was performed from 90 ng RNA samples using 2.2 μL 10× RT buffer, 4.84 μL MgCl2 (25 mM), 4.4 μL dNTPs (10 mM), 1.1 μL random hexamers (50 μM), 1.1 μL oligo(dT)16 (50 μM), 0.44 μL RNase Inhibitor (20 U/μL), 0.55 μL Multiscribe (50 U/μL) in a total volume of 22 μL (final cDNA concentration: 4 ng/μl). Samples were incubated at 25°C for 10 minutes and 42°C for 60 minutes. The reaction was stopped by heating to 95°C for 5 minutes. TaqMan reactions were performed using Applera TaqMan Universal PCR Master Mix (4305719) and TaqMan Rodent GAPDH Control Reagents (4308313). Oligonucleotides were synthesized by Operon Biotechnologies GmbH (Köln, Germany). For *Scn11a *the following sequences were selected: target forward primer: GAGGAATGTGCCGCTGTCA, target reverse primer: CTTTCAGCTTCAGCTTGATCATCTT. The target probe was labeled with FAM: CCATGTGTCTCCGGTAGGCCCTCTG. For Gal the sequences were: target forward primer: CATGCCATTGACAACCACAGA, target reverse primer: TCCTTTCCTCCACCTCCAGTT, target probe labeled with FAM: CCCTCTTGCCTGTGAGGCCATGCT. PCR was performed using the ABI Prism 7900 (Applera) under the following PCR conditions: 2 minutes at 50°C, 10 minutes at 95°C, 40 cycles with 95°C for 15 s and 1 minute at 60°C. PCR was set up as a multiplex PCR using 0.125 μL target probe (50 μM), 0.45 μL target forward primer (50 μM), 0.45 μL target reverse primer (50 μM), 12.5 μL TaqMan 2× PCR Master Mix, 0.25 μL each of primers and probes (TaqMan Rodent GAPDH Control Reagents), 2.5 μL or 1.25 μl cDNA sample in a total reaction volume of 25 μl.

### Microarray expression profiling

First-strand cDNA synthesis was performed using 500 ng total RNA with a 100 pM T7-(dT)24 oligomer (GGCCAGTGAATTGTAATACGACTCACTATAGGGAGGCGG-dT24) according to Baugh et al. [34] and SuperScript II reverse transcriptase following the manufacturer's instructions. Double-stranded cDNA was synthesized and then extracted using phenol-chloroform followed by an ethanol precipitation step. An in vitro transcription reaction was performed with the double stranded cDNA sample using the BioArray High Yield RNA Transcription Labeling kit (Enzo Life Sciences, Farmingdale, NY, USA) according to the manufacturer's instructions. Transcription reactions were incubated at 37°C for 16 h. cRNA was purified using the RNeasy Mini kit protocol for RNA cleanup (Qiagen GmbH, Hilden, Germany) and quantified spectrophotometrically. Mice were excluded if < 12 μg of cRNA was obtained. The biotin-labeled cRNA was fragmented using a RNA fragmentation buffer (200 mM Tris-acetate, 500 mM KOAc, 150 mM MgOAc, pH 8.1). Hybridization and staining on mouse MG430_2 GeneChipsTM (Affymetrix Inc., Santa Clara, CA, USA) was performed according to the manufacturer's instructions.

The microarrays were scanned using a GeneChip 3000 Scanner. The scanned data were analyzed using Resolver v5.1 expression data analysis software (Rosetta Biosoftware, Seattle, WA, USA). Before computing correlations, the array data were filtered for significant expression. The criterion for expression for each gene (i.e. for each transcript included on the array) was a p-value < 0.001 for signal intensity vs. noise level in at least 5 L5DRGs, considering all 5 mouse strains together. Only genes that were expressed above this level were included in the correlation analysis. Means are given ± the standard deviation (SD) unless otherwise indicated.

### Correlational analysis

#### In situ hybridization data

For each target gene the proportion of mRNA-positive neuronal profiles in the 4 DRGs from naive mice and the 4–6 DRGs from nerve injured mice was calculated. The Mann-Whitney U test was used to evaluate the significance of changes for each gene by comparing proportions in the naïve and operated mice (Statistica, StatSoft Scandinavia AB, Uppsala, Sweden). In addition, values from the individual naive mice were averaged to yield a baseline expression level for each gene in each strain (mean percent ± SEM). Change in expression of a given transcript for each nerve injured mouse was calculated by subtracting the mouse's individual expression value from the mean of the naïve mice in the same strain. The values for change in expression were then averaged for each strain and compared across strains using one-way ANOVA. Average change in expression values for all replicate pairs in each of the five strain were converted to ranks (1 to 5) where 1 represents the strain with the smallest magnitude of down-regulation in expression and 5 represents the strain with the largest. Finally, strain average values for regulation were plotted against the strain-specific score for pain sensibility to yield Pearson (parametric) and Spearman (rank) correlation coefficients (r_P _and r_S _; n = 5 data points per plot).

#### Microarray data – Mouse strains based analysis

For each of the five strains L5 nerve injury-induced change in expression for each gene (fold up- or down-regulated) in the L5 and the L4DRGs was calculated. The calculation, log of the ratio of SNL vs. sham expression based on intensity values merged over all animals in each strain, was implemented by the corresponding algorithm in Resolver v5.1. Only transcripts with fold regulation ≥ 1.5 in at least three strains, or ≥ 2 in at least one strain were included for further analysis. Overall, 3927 transcripts met this criterion (3 dpo). For these transcripts, gene expression data for all individuals in each of the five mouse strains was averaged, and the result plotted against strain-specific pain phenotype and r_P _and r_S _were calculated (n = 5 data points per plot). Plots were also made using raw signal intensities from DRGs of operated mice. Pain phenotype was based on the values given in Table 1.

#### Microarray data – Individual mouse analysis

This analysis was carried out for the three strains in which we assessed tactile allodynia in individual mice (AKR, B6 and CBA). Rather than averaging over all SNL operated animals in a given strain, fold regulation of gene expression in the ipsilateral L5 and L4 DRGs was calculated for each DRG, in each individual mouse, in each strain. Specifically, we calculated the log of the ratio of expression intensity in individual SNL operated DRGs vs. the average expression intensity for the corresponding DRGs in the sham operated mice of the same strain (Resolver v5.1). Genes with fold regulation ≥ 1.5 in at least 60% of all SNL operated mice in the three strains, or ≥ 2 in at least 20% of these mice, were used for correlational analysis. 3963 transcripts met this criterion. Data on the expression of individual genes in each mouse were plotted against the behavioral results of the individual mice and r_p _was calculated (n = 26 data points per plot). Plots were also made using raw signal intensities from DRGs of operated mice.

Statistical formalism calls for p-values associated with calculated correlation coefficients r_p _and r_s _to be corrected for multiple testing. As in microarray expression analyses the very large number of comparisons made undermines the ability to declare any given comparison statistically significant. However, the list of genes with high r_p _and r_s _is nonetheless expected to be enriched in candidates whose regulation is related to pain phenotype. The evaluation of enrichment can be enhanced by considering false discovery rates (FDRs) [35]. FDR analysis considers the distribution of r-values and asks whether the number of high values exceeds that expected at random. It then assigns a q-value which indicates the expected number of false discoveries in the set of genes with large r values. The absolute value of r_P _was used in the FDR analysis. Computations were done in the R environment using the packages "stats" and "fdrtool" [36]. A value of q < 0.05 was considered to be significant. Lists of correlated genes were further analyzed according to functional category, both raw categorical distributions and enrichment with respect to the entire mouse genome, using the Gene Ontology Tree Machine (GOTM, [37]) and Ingenuity Pathway Analysis (IPA, Ingenuity Systems, Redwood City, CA, USA; [38]).

## Competing interests

Some of the authors are employees of the commercial firm Sanofi-Aventis. Sanofi-Aventis has intellectual property rights in some of the results published in this paper, and related results not included in the paper. The authors who are employees do not have a significant direct conflict of interests with regard to this paper. However, they would potentially benefit from the success of the company and in this sense might be considered to have an indirect conflict of interests.

## Authors' contributions

Authors MG, SJ, CMW, AMS, HCS, DDP, and JT carried out the surgery, phenotyping hybridization and data analysis associated with the microarray studies. AKP, XJX, ZWH and KF carried out the corresponding tasks associated with the in situ hybridization work. AKP, MG, AD, KF and MD conceived of the project and managed it, carried out statistical, genomic and literature reviews, and drafted the text, which was approved by all authors.

## Supplementary Material

Additional file 1 **Table S1 – Gene transcripts most up- and down-regulated following axotomy**. This Table provides the Affymetrix sequence code, the sequence name and the sequence description of the transcripts with the greatest degree of up- and down-regulation in the L5DRG 3 days following L5 spinal nerve transaction. Data are based on the AKR mouse strain. Click here for file
