# Supplementary material for: Correlational analysis for identifying genes whose regulation contributes to chronic neuropathic pain
Source: Mol Pain. 2009 Feb 19;5:7. doi: 10.1186/1744-8069-5-7 (PMC2649910; doi:10.1186/1744-8069-5-7)
Supplement: Additional file 1 — Table S1 – Gene transcripts most up- and down-regulated following axotomy. This Table provides the Affymetrix sequence code, the sequence name and the sequence description of the transcripts with the greatest degree of up- and down-regulation in the L5DRG 3 days following L5 spinal nerve transaction. Data are based on the AKR mouse strain. [file 1744-8069-5-7-S1.doc]

**Additional files**

**Additional file 1**

**File format: DOC**

**Supplementary Table 1: Gene transcripts most up- and down-regulated following axotomy.**

This Table provides the Affymetrix sequence code, the sequence name and the sequence description of the transcripts with the greatest degree of up- and down-regulation in the L5DRG 3 days following L5 spinal nerve transaction. Data are based on the AKR mouse strain.

| **Sequence Code** | **Primary Sequence Name** | **Sequence Description** | **Accession #** | **Ratio (SNL vs. sham)** | **Fold Change (SNL vs. sham)** | **P-value** |
| --- | --- | --- | --- | --- | --- | --- |
| **up-regulated** | | | | | | |
| 1460663_at | Cckbr | Mus musculus cholecystokinin B receptor (Cckbr), mRNA. | NM_007627 | 43.019 | 43.019 | 0 |
| 1447812_x_at | Flnc | Filamin C, gamma (actin binding protein 280), mRNA (cDNA clone IMAGE:6824907) | AV014577 | 33.556 | 33.556 | 0 |
| 1449201_at | Star | Steroidogenic acute regulatory protein (Star), mRNA | L36062 | 28.598 | 28.598 | 0 |
| 1444687_at | C1ql2 | C1qTNF10 (C1qtnf10) | BB804635 | 25.06 | 25.06 | 0 |
| 1421134_at | Areg | Mus musculus amphiregulin (Areg), mRNA. | NM_009704 | 24.445 | 24.445 | 0 |
| 1425243_at | Cd207 | CD 207 antigen (Cd207), mRNA | AY026050 | 24.389 | 24.389 | 1.27E-34 |
| 1417156_at | Krt1-19 | Mus musculus keratin complex 1, acidic, gene 19 (Krt1-19), mRNA. | NM_008471 | 23.469 | 23.469 | 4.07E-33 |
| 1447836_x_at | 4921525O09Rik | PREDICTED: hypothetical protein LOC74050 [Mus musculus], mRNA sequence | AV312506 | 22.088 | 22.088 | 5.52E-35 |
| 1441350_at | Fgf3 | Fibroblast growth factor 3 (Fgf3), mRNA | AV302620 | 22.066 | 22.066 | 0 |
| 1419127_at | Npy | Mus musculus neuropeptide Y (Npy), mRNA. | NM_023456 | 21.379 | 21.379 | 0 |
| 1419149_at | Serpine1 | Mus musculus serine (or cysteine) peptidase inhibitor, clade E, member 1 (Serpine1), mRNA. | NM_008871 | 21.3 | 21.3 | 6.99E-37 |
| 1454770_at | Cckbr | Cholecystokinin B receptor (Cckbr), mRNA | AV221910 | 19.942 | 19.942 | 0 |
| 1427017_at | Satb2 | Special AT-rich sequence binding protein 2 (Satb2), mRNA | BB104560 | 18.833 | 18.833 | 0 |
| 1424248_at | Arpp21 | Cyclic AMP-regulated phosphoprotein, 21 (Arpp21), transcript variant 1, mRNA | BB159263 | 17.741 | 17.741 | 4.50E-22 |
| 1440635_at | Palld | MKIAA0992 protein | BB534971 | 17.467 | 17.467 | 1.79E-04 |
| 1420433_at | Taf7l | TAF7-like RNA polymerase II, TATA box binding protein (TBP)-associated factor (Taf7l), mRNA | AF285574 | 16.236 | 16.236 | 1.45E-17 |
| 1450610_at | Ucn | Mus musculus urocortin (Ucn), mRNA. | NM_021290 | 16.07 | 16.07 | 0 |
| 1457026_at | Liph | Lipase, member H (Liph), mRNA | BB367422 | 15.054 | 15.054 | 0 |
| 1448061_at | Msr1 | Macrophage scavenger receptor 1 (Msr1), mRNA | AA183642 | 14.851 | 14.851 | 0 |
|  |  |  |  |  |  |  |
| **down-regulated** | | | | | | |
| 1454768_at | Kcnf1 | Potassium voltage-gated channel, subfamily F, member 1 (Kcnf1), mRNA | AV337635 | 0.041 | -24.114 | 0 |
| 1417215_at | Rab27b | BB121269 RIKEN full-length enriched, adult male urinary bladder Mus musculus cDNA clone 9530081E16 3', mRNA sequence. | BB121269 | 0.041 | -24.685 | 1.03E-34 |
| 1421652_at | Htr3b | Mus musculus 5-hydroxytryptamine (serotonin) receptor 3B (Htr3b), mRNA. | NM_020274 | 0.04 | -25.185 | 0 |
| 1446501_at | A830053O21Rik | RIKEN cDNA A830053O21 gene (A830053O21Rik), mRNA | BB271275 | 0.04 | -25.268 | 0 |
| 1441647_at | Fyn | BB307427 RIKEN full-length enriched, adult male corpora quadrigemina Mus musculus cDNA clone B230214I08 3', mRNA sequence. | BB307427 | 0.039 | -25.888 | 0 |
| 1418723_at | Edg7 | Mus musculus endothelial differentiation, lysophosphatidic acid G-protein-coupled receptor 7 (Edg7), mRNA. | NM_022983 | 0.038 | -26.099 | 0 |
| 1434295_at | Rasgrp1 | RAS guanyl releasing protein 1 (Rasgrp1), mRNA | BE691356 | 0.037 | -27.233 | 0 |
| 1438500_at | B230206N24Rik | Cysteine-rich with EGF-like domains 1, mRNA (cDNA clone MGC:36401 IMAGE:5310111) | BE954519 | 0.036 | -27.56 | 0 |
| 1451858_at | Mrgpra2 | MAS-related GPR, member A2 (Mrgpra2), mRNA | AY042192 | 0.036 | -27.734 | 0 |
| 1421400_at | Kcnmb1 | Potassium large conductance calcium-activated channel, subfamily M, beta member 1, mRNA (cDNA clone MGC:13890 IMAGE:3988005) | BB633976 | 0.035 | -28.966 | 0 |
| 1437030_at | Plcd4 | Phospholipase C, delta 4 (Plcd4), mRNA | AV257260 | 0.033 | -29.991 | 0 |
| 1421369_a_at | Mab21l1 | Mus musculus mab-21-like 1 (C. elegans) (Mab21l1), mRNA. | NM_010750 | 0.033 | -30.074 | 5.50E-36 |
| 1453060_at | Rgs8 | Regulator of G-protein signaling 8 (Rgs8), mRNA | AK018337 | 0.033 | -30.124 | 0 |
| 1429922_at | 1429922_at | Mus musculus adult male hypothalamus cDNA, RIKEN full-length enriched library, clone:A230108N10 product:unclassifiable, full insert sequence. | AK020721 | 0.033 | -30.206 | 0 |
| 1450427_at | Chrna6 | Cholinergic receptor, nicotinic, alpha polypeptide 6, mRNA (cDNA clone MGC:25239 IMAGE:4501558) | AW048864 | 0.024 | -41.634 | 0 |
| 1450143_at | Rasgrp1 | RAS guanyl releasing protein 1 (Rasgrp1), mRNA | BB354696 | 0.024 | -41.708 | 9.41E-39 |
| 1420573_at | Hoxd1 | Mus musculus homeo box D1 (Hoxd1), mRNA. | NM_010467 | 0.023 | -42.885 | 0 |
| 1457651_x_at | Rem2 | Rad and gem related GTP binding protein 2 (Rem2), mRNA | BB270375 | 0.019 | -51.969 | 0 |
| 1450426_at | Chrna6 | Cholinergic receptor, nicotinic, alpha polypeptide 6, mRNA (cDNA clone MGC:25239 IMAGE:4501558) | AW048864 | 0.015 | -67.274 | 4.98E-30 |
| 1426121_at | Mrgpra3 | MAS-related GPR, member A3 (Mrgpra3), mRNA | AY042193 | 0.01 | -100 | 0 |
|  |  |  |  |  |  |  |
